# Association of the dietary patterns with the risk of non-alcoholic fatty liver disease among Iranian population: a case-control study

**DOI:** 10.1186/s12937-020-00580-6

**Published:** 2020-06-30

**Authors:** Narges Dehghanseresht, Sima Jafarirad, Seyed Pejman Alavinejad, Anahita Mansoori

**Affiliations:** 1 Department of Nutrition, Faculty of Allied Medical Sciences, Ahvaz Jundishapur University of Medical Sciences, P.O.BOX: 61357-15794, Ahvaz, Iran; 2 Nutrition and Metabolic Diseases Research Center, Ahvaz Jundishapur University of Medical Sciences, Ahvaz, Iran; 3 Research Institute for Infectious Disease of Digestive System, Ahvaz Jundishapur University of Medical Sciences, Ahvaz, Iran

**Keywords:** NAFLD, Fatty liver, Dietary pattern, Factor analysis, Iran

## Abstract

**Background:**

Diet-based recommendations can be developed for preventing and treating non-alcoholic fatty liver disease (NAFLD) after investigating the effects of whole diets on NAFLD. The aim of this study was to identify major dietary patterns and their association with the risk of NAFLD.

**Methods:**

A total of 244 individuals (122 NAFLD patients and 122 controls) participated in this case-control study. The patients with NAFLD were diagnosed by a gastroenterologist. The participants’ dietary intake data were collected using a 147-item semi-quantitive food frequency questionnaire and major dietary patterns were identified by principal component analysis. Adherence to dietary patterns was divided into tertiles and its association with odds of NAFLD was investigated by multivariate logistic regression.

**Results:**

The results showed four major dietary patterns, among which adherence to the “ordinary pattern” was positively associated with NAFLD risk. After adjusting for all confounding factors, individuals in the highest tertile of “ordinary pattern” exhibited a significantly elevated risk of NAFLD compared to the lowest tertile (OR = 3.74, 95%CI = 1.23–11.42, P trend< 0.001). As well as, Individuals in the second and third tertiles of the “traditional pattern” were associated with the risk of NAFLD compared to the lowest tertile (medium vs. lowest tertile OR = 2.37, 95%CI = 1.02–5.53; highest vs. lowest tertile OR = 3.58, 95% CI = 1.48–8.68, P trend< 0.001). The highest tertile of “vegetable and dairy pattern” compared to the lowest tertile was inversely associated with NAFLD risk (OR = 0.23, 95%CI = 0.09–0.58, P trend = 0.02). No significant association was found between “fast food type pattern” and the risk of NAFLD.

**Conclusion:**

A significant association was observed between different dietary patterns and the risk of NAFLD. These results can potentially serve as a dietary strategy for preventing NAFLD in individuals who are at a high risk for progression of NAFLD.

## Background

Non-alcoholic fatty liver disease (NAFLD), the most common worldwide cause of liver disease [1], is identified by an excessive flux of free fatty acids (FFA) and accumulation of triglycerides (TG) in the liver [2]. The prevalence of NAFLD is approximately 25 and 33.9% in the world and Iran, respectively [3, 4]. The NAFLD increases inflammation and mitochondrial dysfunction in the liver that result in hepatic steatosis [5]. It may develop into steatohepatitis, fibrosis, cirrhosis, and hepatocellular carcinoma in some individuals [6]. Moreover, patients with NAFLD have an increased risk of cardiovascular diseases [7]. The common causes of triglyceride accumulation in hepatocytes are insulin resistance, obesity, and, dietary factors among individuals without excessive alcohol consumption, use of steatogenic drugs, or genetic diseases [8, 9]. Indeed, no pharmacological therapy has been confirmed for NAFLD, but lifestyle modifications such as weight loss, dietary change, and increased physical activity are among the first-line treatment for patients with NAFLD [10, 11]. Nutrition not only is a potential factor in pathogenesis of NAFLD, but also plays an important role in its treatment [12]. According to the literature, excessive energy intake or inappropriate diets, such as high carbohydrate or high-fat diet, were associated with the onset and progression of this disease [13, 14]. However, decreased energy intake or adherence to high protein, high monounsaturated, and n-3 polyunsaturated fatty acids (PUFA), and antioxidant intake were reported to decrease the hepatic steatosis [15–17].

Several epidemiological studies investigated the relationship of single nutrients or foods with the risk of NAFLD, but few studies examined the effect of a whole diet on the disease and the results are controversial. For example, although the findings of some studies suggest a positive relationship between a diet rich in carbohydrates and fast foods and the risk of NAFLD [5, 18], Other studies have not found similar results [19, 20]. As well as, some studies reported a dietary pattern full of animal meats increased the risk of NAFLD [21–23], while another study has not reported a significant relationship between high consumption of meat and NAFLD [5]. Also, the results of the effect of fruit consumption on NAFLD risk are inconsistent [5, 21, 24].

Some studies reported a negative association between NAFLD development and diets full of plant foods and fish with less red meat. These food groups are rich in antioxidants, vitamins, minerals, n-3 PUFA, and dietary fibers. However, dietary patterns rich in sugar and fat such as red meat, fast food, sweets, refined grains, and soft drinks had a positive association with the risk of NAFLD [18, 23, 24].

Considering lack of the related information in Iran, dietary patterns of patients with NAFLD were investigated in Ahvaz City, located in the south-west of Iran to identify the major dietary patterns leading to NAFLD.

## Method

### Study participants

This case-control study was conducted from November 2018 to May 2019 among patients who referred to a gastroenterology outpatient clinic for health check. As a result, 122 patients affected by NAFLD and 122 non-NAFLD participants aged 19–70 years were recruited. Exclusion criteria were physical or mental disability, chronic diseases such as diabetes mellitus and liver neoplasm, other liver diseases like viral hepatitis and alcoholic fatty liver, immunodeficiency virus, hepatotoxic or contraceptive drugs use, alcohol consumption of more than 20 g for men and 10 g for women per day, and any type of malignancy [25]. All participants were informed about the study goals and were asked to sign a consent form to enter the study. Furthermore, the study protocol was confirmed by the Ethics Committee in Jundishapur University of Ahvaz based on the ethical guidelines of the 1975 declaration of Helsinki.

The NAFLD was diagnosed by a gastroenterologist considering the elevated alanine aminotransferase (normal range: 29 to 33 IU/l for males and 19 to 25 IU/l for females), elevated aspartate aminotransferase (normal range: 10–40 IU/l for males and 9–32 IU/l for females), and confirmed liver steatosis in the ultrasound examination [26]. The control group members were matched with the patients in terms of their age (in five-year categories), gender (male/female), and body mass index (BMI). The participants were classified as normal-weight, over-weight, and obese according to their BMI of 18–24.9, 25–29.9, and above 30 kg/m^2^, respectively [27]. In addition, all patients underwent an ultrasound examination and no evidence of hepatic steatosis was observed among the control group.

### Measurement of anthropometrics and other variables

The participants’ demographic information including age, gender, ethnicity, marital status, educational level, occupational status, and smoking was collected using a socio-demographic questionnaire. All anthropometric measurements were performed by the same interviewer. The participants’ body weight was measured and recorded to the nearest 0.5 kg in light clothes and without shoes by a digital scale. Height was also measured to the nearest 0.1 cm using a tape meter while the participant was standing in a straight position leaning against the wall with no shoes [28]. The participants’ BMI was also computed by dividing weight in kg by the square of height in meter. The waist circumference (WC) was measured to the nearest 0.1 cm using a tape meter at the midpoint between the lowest rib and the iliac crest. In addition, the hip circumference was measured to the nearest 0.1 cm by a tape meter at the largest circumference of the buttocks. The waist/height ratio (WHtR) and the waist/hip ratio (WHR) were also calculated [29, 30]. For blood pressure measurement were first asked participants to rest for 10 min in a seating position, then blood pressure was measured twice using a standardized sphygmomanometer and the mean value was recorded. Hypertension was defined as systolic pressure > 140 mmHg and diastolic pressure > 90 mmHg or intake of antihypertensive drugs. Physical activity was measured by a validated questionnaire and was expressed as metabolic equivalents hour/day (METs-h/d) in which nine different MET levels were ranged on a scale from sleep/rest (0.9 METs) to high-intensity physical activities (> 6 METs) [31]. The time spent per day in a variety of physical activities was reported by the participant. The time spent in each activity was multiplied by its typical energy expenditure, expressed in terms of metabolic equivalents (METs). The resulting values were added together to yield a MET-hours/day score.

### Dietary assessment

A valid and reliable semi-quantitative 147-item food frequency questionnaire (FFQ) was administered to evaluate the individuals’ usual dietary intake using the standard serving size commonly consumed by Iranians [32–34]. Participants were required to report their consumption frequency of an intended serving of each food item during the last year on a daily, weekly, monthly, or annually basis. Later, the selected frequency category for each food item was converted into a daily intake. Household measures were applied to convert portion size of the consumed foods to grams [35]. Food items were classified into 19 food groups based on their *similarity* in *nutritional composition* and previous studies [36, 37] (Additional file 1). The major dietary patterns were determined by principal component analysis.

### Statistical analysis

Data were analyzed using SPSS (version 25; SPSS Inc., Chicago, IL, USA) by running the student’s t-test for normally distributed variables, Mann Whitney test for non-normally distributed variables, and chi-squared tests for categorical variables. Quantitative and qualitative variables were expressed as mean ± SD and percentage, respectively. The dietary patterns were identified by principal component analysis. Two statistical tests, Bartlett’s test of sphericity, and Kaiser-Myer-Olkin (KMO) measure of sampling adequacy. Bartlett’s test of sphericity should be significant (*p* < 0.05). The KMO index ranges from 0 to 1, with 0.6 suggested as the minimum value for a good factor analysis [38]. In this study sufficiency of the sampling and data was approved by KMO values > 0.6 and *P* ≤ 0.05 for Bartlett’s test sphericity test. There are a number of techniques that can be used to assist in the decision concerning the number of factors to retain. One of the most commonly used techniques is the eigenvalue rule. The eigenvalue of a factor represents the number of the total variance explained by that factor. Using this value, only factors with an eigenvalue of 1.0 or more are retained for further investigation. As well as, another approach that can be used is the scree plot. This involves plotting each of the eigenvalues. Whereby the point at which the graph starts to become horizontal indicates the maximum number of factors to be retained [38]. In the current study, the number of factors (dietary patterns) was determined considering the criteria of eigenvalue> 1.3 and the analysis of the scree plot. For simplification of the data interpretation, orthogonal rotation (varimax) was applied. Food groups with a factor loading of ≥ ±0.3 were included in the analysis. The factor loading is the coefficient of correlation between the food group and the factor [38]. Thus, factor loadings of <│0·3│were not interpreted, as these did not make a significant contribution to the pattern. The patterns were named based on the highest factor loadings on each pattern. Subsequently, dietary patterns were divided into tertiles; where, the first tertile indicated low intake and the third one showed high adherence to the dietary pattern. The association between tertiles of 4 dietary patterns and risk of NAFLD was calculated by odds ratio (OR) and the 95% confidence intervals (CIs) using multivariable logistic regression. In this regard, three models of logistic regression were assessed; model 1 was crude, model 2 was adjusted for age, gender, energy intake, and BMI, and model 3 was further adjusted for smoking, educational status, and physical activity.

## Result

Table 1 shows the participants’ demographic characteristics. Dietary information of one of the NAFLD group members was excluded because this patient’s energy intake was more than 3 standard deviations from the mean on the log-transformed scale. Finally, a total of 243 participants were included in the analysis. Patients have significantly higher WC (*p* = 0.001), WHtR (*p* < 0.001), WHR (p < 0.001), and energy intake (p < 0.001). Moreover, patients were significantly less educated and smoked more frequently than the controls (*p* < 0.05).

**Table 1:** Characteristics of participants

Variables	NAFLD (*n* = 121)	Control (*n* = 122)	*P*-value^a^
Age (year), Mean ± SD	42.95 ± 11.46	42.51 ± 11.52	0.71^b^
Sex			0.95^c^
Male	57 (47.1%)	58 (47.5%)	
Female	64 (52.9%)	64 (52.5%)	
Weight, Kg	81.78 ± 13.12	80.76 ± 13.28	0.55^d^
Height, cm	165.53 ± 10.16	165.97 ± 9.19	0.68^b^
BMI, kg/m^2^	30.53 ± 5.04	29.32 ± 4.49	0.08^b^
Waist circumference, cm	102.86 ± 10.78	98.08 ± 10.55	0.001^d^
Hip circumference, cm	105.91 ± 7.59	105.35 ± 7.41	0.58^d^
WHtR	0.62 ± 0.07	0.59 ± 0.07	< 0.001^d^
WHR	0.95 ± 0.07	0.92 ± 0.08	0.002^b^
Systolic blood pressure, mmHg	124.09 ± 12.29	121.02 ± 14.45	0.32^b^
Diastolic blood pressure, mmHg	81.35 ± 6.96	80.14 ± 6.44	0.28^b^
Total energy intake, kcal	4122.76 ± 1624.85	3178.60 ± 936.18	< 0.001^d^
Met (Hour/day)	34.11 ± 5.87	35.94 ± 7.88	0.14^b^
Marital status			0.72^c^
Married	105 (86.8%)	102 (83.6%)	
Bachelor	16 (13.2%)	20 (16.4%)	
Educational status			0.002^c^
Illiterate	14 (11.6%)	2 (1.6%)	
Elementary	36 (29.8%)	30 (24.6%)	
Diploma	34 (28.1%)	31 (25.4%)	
College	37 (30.6%)	59 (48.4%)	
Smoke			0.04^c^
Yes	12 (9.9%)	4 (3.3%)	
No	109 (90.1%)	118 (96.7%)	

^a^*P*-value < 0.05 was considered significant

^b^*P*-value based on the Mann-Whitney test

^c^*P*-value based on the chi-squared test

^d^*P*-value based on the t-test

*NAFLD* nonalcoholic fatty liver disease; *BMI* body mass index; *WHtR* waist to height ratio; *WHR* waist to hip ratio; *MET* the metabolic equivalent of tasks

Dietary information of participants was analyzed by principal component analysis and four dietary patterns were distinguished based on the eigenvalue > 1.3 and scree plot analysis. The first pattern was named “ordinary” pattern, identified by high intakes of sweets, oils, fruits, white meats, refined grains, tea and coffee, salt, biscuits, snacks, red, and organ meats. The second pattern was named the “fast-food type” identified by a high intake of sauces, pickles, fast foods, soft drinks, snacks, and biscuits. The third dietary pattern was labeled as “traditional pattern” characterized by high amounts of red and organ meats, dairy products, condiments, salt, tea and coffee, and low intake of fruits. The fourth pattern was named “ vegetable and dairy” characterized by high amounts of vegetables, whole grains, legume and nuts, and dairy products. The list of food groups and their factor loadings are included in Table 2. These dietary patterns explained 16.35, 12.57, 8.73, and 8.67% of the total variance, respectively.

**Table 2:** Rotated factor loading matrix for the four dietary patterns

Food groups	Dietary patterns			
	Ordinary	Fast food type	Traditional	Vegetables and Dairy
Sweets	0.72	–	–	–
Oils	0.65	–	–	–
Fruits	0.63	–	−0.33	–
White meats	0.59	–	–	–
Refined Grains	0.57	–	–	–
Tea and Coffee	0.48	–	0.32	–
Salt	0.47	–	0.31	–
Biscuits	0.39	0.37	–	–
Sauces	–	0.76	–	–
Pickles	–	0.62	–	–
Fast foods	–	0.60	–	–
Soft drinks	–	0.57	–	–
Snacks	0.39	0.56	–	–
Condiments	–	–	0.79	–
Red & organ Meats	0.36	–	0.49	–
Vegetables	–	–	–	0.67
Whole grains	–	–	–	0.60
Legume and Nuts	–	–	–	0.58
Dairy products	–	–	0.34	0.47
Total variance explained	16.35	12.57	8.73	8.67

Values less than |0.3| were excluded. Bartlett’s test of sphericity:964, significance < 0.001; Kaiser–Meyer–Olkin test = 0.74

Table 3 indicates the association between tertiles of dietary patterns and the risk of NAFLD. In this regard, three models of logistic regression were assessed; model 1 was crude, model 2 was adjusted for age, gender, energy intake, and BMI, and model 3 was further adjusted for smoking, educational status, and physical activity. After adjusting for all confounding factors (model 3), individuals in the highest tertile of “ordinary pattern” exhibited an elevated risk of NAFLD compared to the lowest tertile (medium vs. lowest tertile: OR = 1.71, 95%CI = 0.71–4.11; highest vs. lowest tertile: OR = 3.74, 95%CI = 1.23–11.42), and there was a significant dose-response relationship (P trend< 0.001). As well as, Individuals in the second and third tertiles of the “traditional pattern” were associated with the risk of NAFLD compared to the lowest tertile (medium vs. lowest tertile OR = 2.37, 95%CI = 1.02–5.53; highest vs. lowest tertile OR = 3.58, 95% CI = 1.48–8.68), and there was a significant dose-response relationship (P trend< 0.001). The highest tertile of “vegetable and dairy pattern” compared to the lowest tertile was inversely associated with NAFLD risk (OR = 0.23, 95%CI = 0.09–0.58), and the test for trend was significant (P trend = 0.02). No significant association was found between “fast food type pattern” and the risk of NAFLD.

**Table 3:** Odds ratio and 95% confidence intervals for the association between dietary patterns and NAFLD

	T1	T2	T3	P-trend
Ordinary pattern				
Model 1	1	2.95 (1.45–6.03)	11.86 (5.36–26.21)	< 0. 001
Model 2	1	2.14 (0.97–4.75)	4.55 (1.66–12.46)	< 0.001
Model 3	1	1.71 (0.71–4.11)	3.74 (1.23–11.42)	< 0.001
Fast food type pattern				
Model 1	1	0.80 (0.39–1.62)	1.01 (0.50–2.05)	0.19
Model 2	1	0.82 (0.38–1.81)	0.76 (0.30–1.93)	0.24
Model3	1	0.91 (0.39–2.14)	0.72 (0.26–1.96)	0.19
Traditional pattern				
Model 1	1	2.12 (1.04–4.33)	3.03 (1.41–6.53)	< 0.001
Model 2	1	2.57 (1.19–5.54)	3.78 (1.66–8.65)	< 0.001
Model 3	1	2.37 (1.02–5.53)	3.58 (1.48–8.68)	< 0.001
Dairy and vegetable pattern				
Model 1	1	0.79 (0.39–1.59)	0.41 (0.20–0.86)	0.02
Model 2	1	0.59 (0.28–1.25)	0.21 (0.09–0.49)	0.005
Model 3	1	0.60 (0.26–1.37)	0.23 (0.09–0.58)	0.02

Model 1: crude

Model 2: adjusted for age, sex, BMI and energy intake

Model 3: further adjusted for smoking, educational status, and physical activity

## Discussion

In this case-control study, four major dietary patterns were identified; “ordinary pattern”, “fast food type pattern”, “traditional pattern”, and “vegetable and dairy pattern”. The findings showed that “ordinary” and “traditional” patterns increased the risk of NAFLD significantly, while the “vegetable and dairy pattern” had an inverse association with NAFLD in our study population. However, no significant association was observed between the “fast food type pattern” and the risk of NAFLD. To the best of our knowledge, this is the first study on the association between dietary patterns and NAFLD in Ahvaz City.

The “ordinary pattern” was characterized by a high intake of “sweets, oils, fruits, white meats, refined grains, tea and coffee, salt, Biscuits, snacks, as well as red and organ meats”. Most food groups in this dietary pattern contain high amounts of carbohydrates especially refined sugars, which enhance de novo fatty-acid synthesis in the liver [39].

The “fast food type pattern” was characterized by high consumption of “fast foods, pickles, sauces, soft drinks, snacks, and biscuits”. Although most related studies reported a positive relationship between fast food patterns and the risk of NAFLD [5, 18], we found no association between this dietary pattern and NAFLD.

The “traditional pattern”, characterized by a high intake of condiments, red and organ meats, dairy products, salt, as well as tea and coffee, had a direct association with NAFLD. This study was conducted in Ahvaz City, located in the south-west of Iran. In this region, high consumption of red and organ meats (in the form of kebabs) and the dairy product (especially high-fat dairy) is common. Consumption of condiments and hot beverages (tea and coffee) is also part of the food habits in this area.

The “vegetable and dairy pattern” was characterized by high intakes of “vegetables, whole grains, legume and nuts, and dairy products. We found that high adherence to this pattern reduced the risk of NAFLD, which is probably due to the high amounts of vitamins, minerals, and fiber.

The “ordinary pattern” adherence was associated with high intakes of energy-dense foods with relatively high amounts of fat, animal protein, and refined sugars. Some foods in this dietary pattern, including refined grains, white bread, biscuits, and sweets, led to a rapid increase in postprandial plasma glucose, insulin concentration, and diabetes [39]. Such high glycemic index foods increase hepatic steatosis, especially in insulin-resistant participants [40]. Jia et al. also reported that a high carbohydrate/sugar pattern was associated with NAFLD in women [24]. Our results are in line with the finding of another study in Japan over a population with high intake of carbohydrate, especially sweets. Their results showed a higher risk of NAFLD [41]. However, Chung et al. did not find any relationship between high adherence to the carbohydrate diet and the risk of NAFLD [19]. Our findings confirm results of the studies suggesting that a high fruit diet is associated with NAFLD [21, 42]. Fruits are rich in simple carbohydrates particularly fructose. Fructose has a role in the pathophysiology of NAFLD and high intake of fructose leads to de novo lipogenesis, lipid accumulation, and steatosis in the liver. Moreover, chronic consumption of fructose enhances the hepatic inflammation and oxidative stress, which are responsible for progression of the hepatic disease [43]. Nevertheless, fruits had a negative loading in “traditional pattern”. A possible explanation could be that moderate consumption of fruits due to the presence of fiber, antioxidants, and vitamins can have a protective effect on the fatty liver [44–46]. While excessive fruit consumption due to having simple sugars (especially fructose) can play a role in the Fat accumulation in the liver.

Some previous studies suggested that a “fast food pattern” or a “western diet” was associated with NAFLD [5, 18]. A western diet, characterized by high intakes of fried foods, red and processed meat, refined grains, snacks, sauces, and soft drinks was prospectively associated with NAFLD risk in adolescents [18]. Kalafati et al. also reported similar results about fast food dietary pattern and NAFLD risk [5]. However, we found no significant association between the fast-food pattern and risk of NAFLD. In studies that have reported a positive relationship between fast food and NAFLD, the mean age of the participants is usually lower than in our study. Also, in these studies, a Western or fast food dietary pattern is usually associated with high consumption of refined grains, sweets,and red meat, which consumption of these food groups may have a synergistic effect on the risk of NAFLD, while in our study the fast food pattern does not include food groups that mentioned above [18, 21]. This finding can be due to the differences in dietary patterns ethnicities, cultural groups, and gender. Moreover, dietary patterns may vary over time because of personal preferences and availability of foods [9].

Although meats, such as red meat and visceral meat, are highly loaded with “ordinary pattern” and “traditional pattern”, very few studies have investigated the association between high protein dietary patterns and NAFLD. According to Zelber Sagi et al., all types of meats were significantly associated with an increased risk of NAFLD [22]. This association can be justified by mentioning that high protein intake is associated with insulin resistance and glucose intolerance, which might even increase the incidence of type-2 diabetes [47, 48]. Furthermore, Saturated fatty acids in red and visceral meats lead to an increase in trans-10, cis-12 conjugated linoleic acid, which promotes stress in the endoplasmic reticulum and also increases apoptosis, which is involved in NAFLD pathogenesis [49, 50]. Another possible explanation is that a higher intake of iron and in particular, heme-iron may play a role in the pathogenesis of NAFLD by increasing oxidative stress [51]. The “traditional “and “ordinary” patterns were high in salt, but the findings of the studies are controversial about the role of sodium in NAFLD. A study among a Korean population reported that high salt dietary pattern was associated with increased risk of NAFLD, while another study in the Chinese population detected no significant association between a high salt dietary pattern and NAFLD [23, 52]. Consequently, more studies are required to clarify this association. As well as, the consumption of tea and coffee is also high in the “ordinary” and “traditional” patterns. Some previous studies have reported a potential protective role of coffee (due to its caffeine and antioxidants content) [53–55] and green tea (due to its catechins content) consumption [56] against NAFLD. Consumption of tea and coffee per se may not have a positive relationship with NAFLD, but consumption of these beverages in the “ordinary” and “traditional” patterns along with an inappropriate diet for NAFLD (for example high consumption of red meat, snacks, sweets and biscuits in the ordinary pattern and red meat In the traditional pattern and also low consumption of vegetables, whole grains, legumes) has led to an increased risk of NAFLD.

Yang et al. reported an inverse association between a “grain and vegetable pattern” and NAFLD and mentioned that this association was independent from the confounding factors such as age, gender, BMI, and physical activity [23]. Another study in a Lebanese population also showed that a “traditional Lebanese” diet consisting of vegetables and legumes had a negative association with the odds of NAFLD [21]. These results confirmed our findings by maintaining that a dietary pattern rich in vegetables, legume, and nuts could reduce the risk of NAFLD. This protective effect against NAFLD might be because of the high fiber content in this pattern.

This study has some strengths. This is the first study over the association between major dietary patterns and the risk of NAFLD in Ahvaz City, Iran. Potential confounding factors were identified and controlled in the analysis. Moreover, we used a semi-quantitative FFQ designed for the Iranian population, which resulted in a better representation of the participants’ dietary habits.

Some limitations also exist that should be considered. First, regarding the case-control design of this study, it could not confirm a causal relationship between dietary patterns and NAFLD. Second, the application of an FFQ for data collection may overestimate the participants’ energy intake, which is a great risk. Third, recall bias exists because of the self-reporting nature of the questionnaire.

## Conclusion

The results display that high adherence to the “ordinary” and “traditional” dietary patterns increase the risk of NAFLD, while adherence to a “vegetable and dairy” pattern has a negative association with NAFLD in Ahvaz population. The findings can be served as a dietary strategy for preventing and treating NAFLD. Nevertheless, more studies are required to confirm our results.

## Supplementary information

**Additional file 1.** Food list of 19 food groups in the FFQ of the present study

## Data Availability

The datasets used and/or analyzed during the current study are available from the corresponding author on reasonable request.

## Abbreviations

NAFLD: Nonalcoholic fatty liver disease
FFA: Free fatty acid
TG: Triglyceride
PUFA: Polyunsaturated fatty acid
BMI: Body mass index
WC: Waist circumference
WHtR: Waist to height ratio
WHR: Waist to hip ratio
MET: The metabolic equivalent of tasks
FFQ: Food frequency questionnaire
OR: Odds ratio
CI: Confidence interval
