# Supplementary material for: Association of the dietary patterns with the risk of non-alcoholic fatty liver disease among Iranian population: a case-control study
Source: Nutr J. 2020 Jun 30;19:63. doi: 10.1186/s12937-020-00580-6 (PMC7329390; doi:10.1186/s12937-020-00580-6)
Supplement: Supplementary file 1 — Additional file 1. Food list of 19 food groups in the FFQ of the present study [file 12937_2020_580_MOESM1_ESM.docx]

Appendix 1: food list of 19 food groups in the FFQ of the present study.

| Food group | Food item |
| --- | --- |
| Refined grains | Spaghetti, cooked rice, white breads, baguette, Boiled potato |
| Whole grains | barely, corn |
| Snacks | fried potato, Chips, pofak |
| Legumes and nuts | Lentils, beans, peas, soy beans, Peanuts, almonds, walnuts, pistachios, hazelnuts, seeds |
| White meats | Baked Chicken, baked fish, canned fish, baked eggs |
| Red and organ meats | Beef meat, sheep meat, Organs meat of sheep: Leg, head, (heart, liver, kidney) |
| Fast foods | Pizza, hamburger or sausage |
| Dairy products | High-fat milk, high-fat yogurt, Low-fat milk, Low-fat yogurt, doogh, cheese, ice cream |
| vegetables | All types of fresh, cooked and dried vegetables. |
| Fruits | All types of fresh fruits, dried fruits, dates, fresh fruit juices. |
| Oils | Olive oil, sesame oil, canola or sunflower oil, Vegetable hydrogenated oil, animal oil, butter, margarine, cream, olive |
| Pickles | Sour and salty pickles |
| Sauces | mayonnaise, ketchup |
| condiments | Condiments |
| Salt | Salt |
| Soft drinks | Carbonated Sugar-sweetened soft drinks, industrial fruit juices |
| Biscuits | Biscuits, crackers, cakes |
| Sweets | sugar, honey, jam, candy, halva, chocolates, Candied fruit, cookies |
| Coffee and tea | Coffee, tea |
